# Toxicological safety evaluation of Qin-Zhi-Zhu-Dan formula in rats during the treatment and recovery periods

**DOI:** 10.3389/fphar.2022.987997

**Published:** 2022-08-25

**Authors:** Wenxiu Xu, Dan Chen, Zehan Zhang, Shuling Liu, Congai Chen, Chunyan Sun, Wenchao Ni, Xiangdong Kang, Guojiao Shang, Xueqian Wang, Fafeng Cheng, Qingguo Wang

**Affiliations:** ^1^ School of Traditional Chinese Medicine Department, Beijing University of Chinese Medicine, Beijing, China; ^2^ Dongzhimen Hospital, Beijing University of Chinese Medicine, Beijing, China

**Keywords:** sub-chronic toxicity, scutellaria baicalensis extract, gardenia extract, pulvis fellis suis, reversibility study, organ coefficient, biochemical

## Abstract

**Background:** Qinzhi Zhudan Formula (QZZD), optimized from Angong Niuhuang Wan, consists of *Radix Scutellariae*, *Fructus Gardeniae* and *Pulvis Fellis Suis*. We had investigated the neuroprotective effects of QZZD and its active components, and demonstrated that it could treat cerebral ischemia and dementia through multiple pathways and mechanisms. Nevertheless, toxicological data on this formula still remains limited. In the study, we sought to examine the toxicological effects of QZZD during the treatment and recovery periods.

**Methods:** We investigated potential toxicities of QZZD in Sprague-Dawley (SD) rats *via* 28-day gavage administration. SD rats were randomly divided into control group and treatment groups of A (0.5 g/kg/d QZZD), B (1.5 g/kg/d QZZD), and C (5.0 g/kg/d QZZD). The 56-day course includes treatment period (administration with water or QZZD once a day for 28 consecutive days) and recovery period (28 days). The rats received daily monitoring of general signs of toxicity and mortality, as well as weekly determination of body weight and food consumption. Moreover, the complete blood cell count, biochemistry, coagulation, and urine indicators, organ weights, and histopathological report were analyzed respectively at the end of the treatment and recovery periods.

**Results:** There was no death related to the active pharmaceutical ingredients of QZZD during the treatment period. The maximum no observed adverse effect level (NOAEL) was 0.5 g/kg/d, which is approximately 16.7 times of the equivalent dose of clinical dose in rats. In group TB (1.5 g/kg/d QZZD) and TC (5.0 g/kg/d QZZD), there were adverse effects of blue coloring of tail skin, weight loss, a significant increase of total bilirubin (TBIL), blackening of liver and kidney in gross examination, hyperplasia of bile duct and karyomegaly of hepatocytes in histopathological examination. Besides, in females rats, the food consumption was reduced, while in male rats, there was decrease in triglycerides (TG) and slight increase in white blood cell (WBC) count and neutrophils. In group TC (5.0 g/kg/d QZZD), the indicators of red blood cell (RBC) count, hemoglobin (HGB) and hematocrit (HCT) were decreased slightly, while the platelet count (PLT) was increased. However, these changes were not considered to be toxicologically significant because they resolved during the recovery period.

**Conclusion:** Overall, QZZD exhibited a good safety profile. The maximum no observed adverse effect level was 0.5 g/kg/d, and no target organs toxicity were identified. The present findings might confirm the safety of QZZD in clinical practices.

## Introduction

Traditional Chinese medicine (TCM) has been widely utilized worldwide for thousands of years. TCM is well-recognized for its ability to treat multiple ailments and promote health (Alam et al., 2016). In recent years, natural products have received increasing attention given their low toxicity and high efficacy (Gao et al., 2018; Qi et al., 2020). Chinese herbs and extracts have become a research hotspot for their therapeutic and cytoprotective characteristics for treating neurodegenerative diseases in human beings (Gaire et al., 2014), especially in treating the etiology and pathogenesis of vascular dementia. Overwhelming evidence substantiates the pharmacological activities of the three components (at a ratio of 10:6:1.5) of QZZD: *Radix Scutellariae*, *Fructus Gardeniae* and *Pulvis Fellis Suis* (Gaire et al., 2014; Zhang et al., 2017; Ma et al., 2020; Rui et al., 2020).

*Radix Scutellariae* is the most widely used herb for neuroprotection in TCM. The major phytochemicals isolated from S. baicalensis are flavonoids, glycosides and their glucuronides, such as baicalin, baicalein, and wogonin (Gaire et al., 2014), with good safety and therapeutic effect against cerebral ischemia. *Radix Scutellariae* generally listed in treatment of cerebral ischemia’s traditional formulas including NiuHuangQingXinWan, Angong Niuhuang Boluses, and Huanglian jiedu decoction, and is also the core component of QZZD. Modern studies have shown that *Radix Scutellariae* has antagonistic effects on neuronal cell death and neurodegeneration due to glutamate toxicity, oxidative cascade, inflammation, and apoptosis (Gaire et al., 2014). Furthermore, we found that baicalin could inhibit cerebral ischemia-induced activation of the NFκB/CCL2/CCR2 pathway via targeting NFκB activation and CCL2/CCR2 interaction. *In vitro*, baicalin significantly inhibited the secretion of NO, IL6, TNFα, and CCL2 in macrophages and promoted the secretion of IL13, IFN -γ, and IL-1α (Xu et al., 2022).

*Fructus Gardeniae* (FG) is the dried fruit of *Gardenia jasminoides Ellis* (GjE), which belongs to the family Rubiaceae and is an outstanding traditional medicinal plant used for medicine and food. FG is a traditional Chinese medicine that has been originally recorded in the “Shennong Bencao Jing” and listed in each version of “Chinese Pharmacopoeia”, with the effects of “discharging fire, eliminating vexation, reducing fever and causing diuresis”, cooling blood to remove pathogenic heat. (Chinese Pharmacopoeia Commission, 2010). Approximately 162 chemical compounds have been isolated and identified from FG. Previous studies have reported various pharmacological properties, including beneficial effects on the nervous, cardiovascular and digestive systems, hepatoprotective activity, antidepressant activity, and anti-inflammatory activity (Chen et al., 2020). Studies have shown that *Gardenia jasminoides* extract (GJE) may play a protective effect in improving chronic cerebral ischemia based on its antioxidant properties by scavenging free radicals, reducing NO toxicity and AChE activity in brain neurons (Zhang et al., 2017).

*Pulvis Fellis Suis* (PFS), named “Zhu Danfen” in China, was first listed in the 2000 version of “Chinese Pharmacopoeia” (Shi et al., 2018). According to the Chinese Pharmacopoeia 2020, PFS has a bitter taste and cold nature and belongs to the liver, gallbladder, lung and large intestine channels. It has the functions of clearing heat, moistening dryness, relieving cough, relieving asthma and detoxifying (Chinese Pharmacopoeia Commission, 2020). PFS is rich in substances, including bile acids, bile pigments, mucoproteins, lipids and inorganic substances (He et al., 2017). Modern pharmacological research indicates that these compounds exert diverse biological activities, such as antitussive, anti-inflammatory, antibacterial, cholagogic, antibacterial, anti-tumor and other pharmacological effects (He et al., 2017; Chen et al., 2021), and are clinically used to treat whooping cough, asthma, hepatitis, vaginitis, intrahepatic bile duct stones, intestinal diseases, etc. (He et al., 2011). Our previous study showed that cholalic acid components have a good anti-cerebral ischemic effect. For instance, cholic acid (CA) exerted protective effects on the neurovascular unit (NVU), mediated by increased release of BDNF and further stimulating the BDNF-TrkB-PI3K/Akt and BDNF-TrkB-MAPK/Erk signaling pathways during OGD/R-induced injury (Li et al., 2020).

Our previous research also showed that QZZD could significantly improve learning memory and spatial exploration disorders in Alzheimer’s and vascular dementia rat models (Liu et al., 2022). Additionally, QZZD could reduce neuroinflammation by regulating the TNF Receptor1-ERK1/2-NF-κBp65 pathway and reducing the expression of the β-amyloid protein in APP/PS1 double-transgenic AD mice (unpublished data). Repeated dose toxicity studies play an indispensable role in preclinical evaluation of safety, whose purpose is to predict possible toxicities in human body. It can help to determine no observed adverse effect level (NOAEL) and first-in-human (FIH) dose, which will provide a safe dose range for subsequent application of QZZD in clinical trials (Gua et al., 2014)However, few studies have hitherto investigated QZZD toxicological safety, emphasizing a lack of evidence for further clinical translation.

In the present study, we aimed to evaluate the subchronic toxicity of QZZD. Rats were administered with 0.5, 1.5, and 5.0 g/kg/d of QZZD for 4 weeks, followed by 4 weeks of recovery, and all relative parameters were monitored during these 8 weeks. Collectively, our findings provide the foothold for applying QZZD in clinical therapy and reliable experimental data for its rational and safe application.

## Materials and methods

### Preparation of qinzhi zhudan formula

QZZD was provided by the China Academy of Chinese Medical Sciences and accredited by expert pharmacognosists. The three medicinals (*Radix Scutellaria*, *Fructus Gardeniae* and *Pulvis Fellis Suis*) were mixed at a ratio of 10:6:1.5. The extraction process and quality control were implemented according to our previous study. *Radix Scutellaria* was decocted with water 12 times of its volume twice, 1 h each time. The decoctions were combined and filtered. The filtrate was concentrated to a relative density of 1.05–1.10, and 2 mol/L hydrochloric acid solution is added at 80°C to adjust the pH value to 1.0–2.0. It was then kept warm for 30 min before centrifuged at 4000 rpm for 10 min. The sediment was washed with a small amount of 50% ethanol that was then evaporated. Then it was dried under 60°C under reduced pressure before use. *Fructus Gardeniae* was grinded and extracted at room temperature for 3 times with 75% ethanol 10 times of its volume, 8 h each time. The solution was combined before ethanol recovery at reduced pressure below 60°C. Then the concentrated solution is spray dried before use. *Pulvis Fellis Suis* was mixed with the above two extracts and kept refrigerated at −20°C until use. Chromatography with tandem mass spectrometry (LC-MS-MS) was used in a previous study to analyze the composition of the QZZD and identified baicalin, geniposide, cholic acid, hyodeoxycholic acid, and chenodeoxycholic acid. The results of QZZD-UHPLC-QE-MS are shown in Supplementary Tables S1, S2.

### Chemicals and agents

*Radix Scutellariae* and Fructus Gardeniae were purchased from Anxing Chinese Herbal Medicine Co., Ltd. (No. 200901, China). *Pulvis Fellis Suis* was purchased from Shengtai Chinese Herbal Medicine Co., Ltd. (No. 190901, China). Xylazine Hydrochloride Injection was purchased from Jilin Hualu Mu Animal Health Products Co., Ltd. (No. 20201118). Zolelil ^®^50 was obtained from Virbac Co., Ltd. (No.7XDYA). Tropicamide was supplied by Shandong Doctor Renfreida Pharmaceutical Co., Ltd. (No.20073001/20102901, Jinan, Shandong province, China).

### Animals

A total of 144 Sprague-Dawley (SD) rats of both sexes (specific pathogen-free (SPF) grade) (males weighing 273.4–333.2 g and females 184.2–233.8 g) were purchased from Beijing WeiTongLiHua Laboratory Animal Technology Co., Ltd. The rats were fed with a standard rodent diet (SPF grade, supplied by Beijing Keao Xieli Feed Co. Ltd., Beijing, China) and water (Conform to GB14925-2010, GB5749-2006 standards) *ad libitum* in a Good Laboratory Practice (GLP) center, approved by the China Food and Drug Administration (CFDA) under controlled conditions [temperature (23 ± 3°C) with a relative humidity of 40.10%–76.89%, and maintained under a 12/12-h light/dark cycle]. The cage beddings and water bottles were cleaned daily. The animals were allowed 1 week of acclimatization before the beginning of experimental procedures. The standards of the feeding environment were based on the People’s Republic of China national standard (GB14925-2010), and the Honeywell EBI R430.1 automatic control system was used.

### Grouping and drug administration

An ear tag was attached to each rat with a unique identification number. Cage cards were made according to the animal numbers in Table 1. The determination of dose of QZZD in this study was in accordance with Technical Guidelines for Repeated Dose Toxicity Studies (2014) issued by China Food and Drug Administration (CFDA). To ensure animal welfare and the feasibility of gavage, 5.0 g/kg/d was regarded as a high-dose concentration, and the maximum concentration for gavage was set to 250 mg/ml QZZD. The animals were randomly divided into a control group (CN) and three experimental groups (TA, TB, and TC) according to the QZZD dosage. There were 18 female and 18 male rats in each group. Rats in the TA, TB, and TC groups were administered with 0.5 g/kg/d, 1.5 g/kg/d, and 5.0 g/kg/d QZZD, hence termed as low, middle, and high-dose groups, respectively. The experimental group’s dosage was 20 ml/kg, and the control group received the same volume of double distilled water.

**TABLE 1:** Group grouping and identification of test animals.

Group number	Group	Number		Animal number		Cage number		Necropsy time	Cage card color
		♀	♂	♀	♂	♀	♂		
1	CN	18	18	1101–1112	1201–1212	1–4	7–10	end of administration	white
				1113–1118	1213–1218	5, 6	11, 12	end of the recovery period	
2	TA	18	18	2101–2112	2201–2212	13–16	19–22	end of administration	green
				2113–2118	2213–2218	17, 18	23, 24	end of the recovery period	
3	TB	18	18	3101–3112	3201–3212	25–28	31–34	end of administration	yellow
				3113–3118	3213–3218	29, 30	35, 36	end of the recovery period	
4	TC	18	18	4101–4112	4201–4212	37–40	43–46	end of administration	red
				4113–4118	4213–4218	41, 42	47, 48	end of the recovery period	

Animal numbers 4110 and 4111 were dead at D15 and D9, respectively.

**FIGURE 1:**
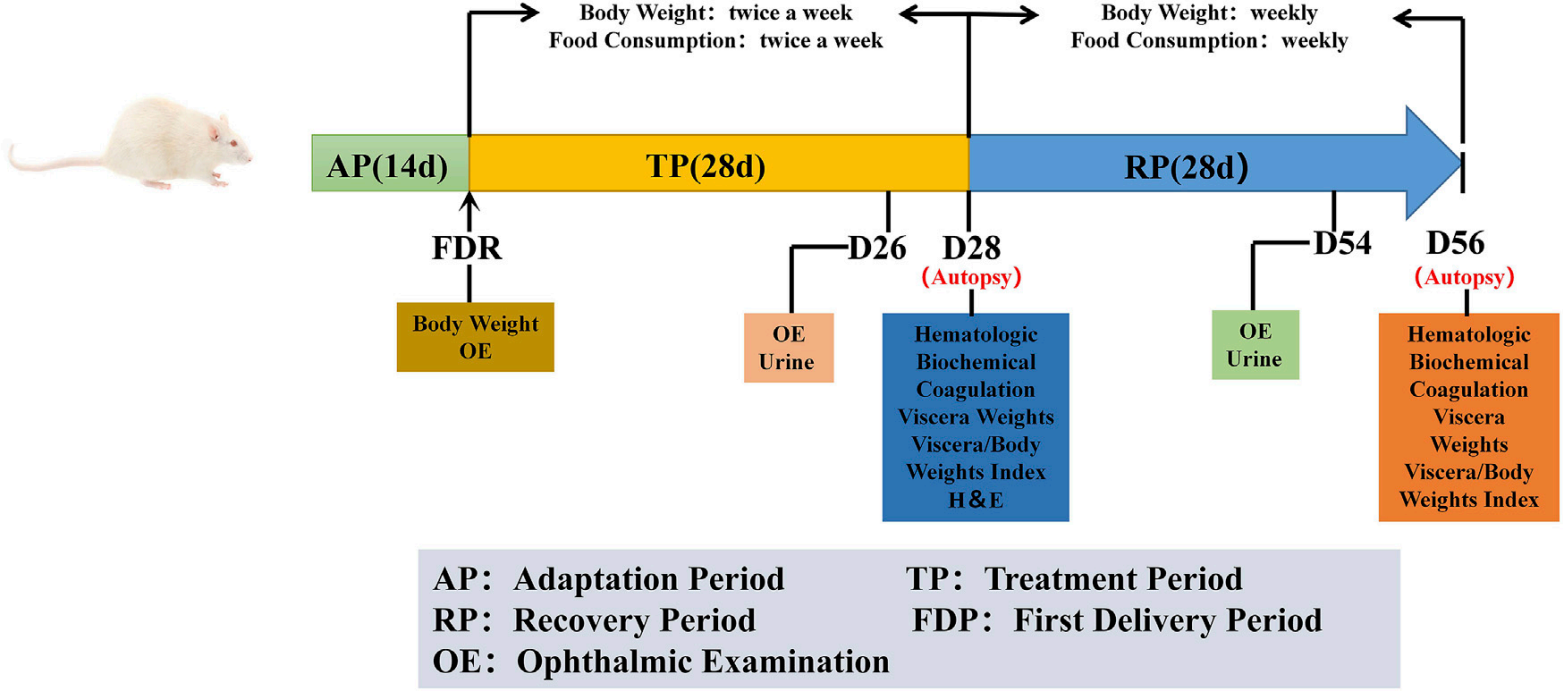
Representative experimental scheme. ‘AP’ indicates 14 days of suitability rearing before the experiment; ‘FDR’ was defined as trial day 1 (D1), body weight and ophthalmic examinations were performed. The whole experiment lasted for 56 days, it was divided into treatment period (TP) and recovery period (RP). During the trial, cage observation daily; detailed clinical signs observation, body weight and food consumption were performed weekly. Ophthalmic examination and urine examination are performed before the end of the treatment and the recovery periods (D26 and D54). Hematologic, biochemical and coagulation examinations were tested at the end of the treatment period and the recovery period (D28 and D56), and necropsy, organ weight and histopathological examination were performed after the animals were euthanized.

At the end of the treatment period, 12 male and 12 female rats from each group were sacrificed for analyses of the complete blood count, biochemistry, and urine indicators, major organ weights, and histopathology of reproductive organs. The remaining rats in each group, including six females and six males, were given free access to food and water for another 4 weeks (5–8 weeks, named recovery period) until the end of the experiment. They were sacrificed at the end of the recovery period, and the above-mentioned parameters were assessed. A representative experimental scheme is shown in Figure 1.

### Clinical signs observation

Cage-side observations for apparent signs of toxicity or injury (behavior, mental status, gland secretion, feces characteristics, genitals, and death) were conducted twice a day after daily drug exposure, and once experimental animals developed toxic symptoms, the observation frequency was increased. During the recovery period, the observation was performed once a day.

Detailed clinical observations were made before grouping and once a week during administration until the end of the trial. If the detailed clinical observation coincided with the cage-side observation time, the detailed clinical observation replaced the cage-side observation in this period.

### Body weight and food consumption

The body weight in all groups was measured once during the adaptation period (when grouping) and then measured twice per week (D3, D7, D10, D14, D17, D21, D24, D28) during the treatment period and then measured weekly (D35, D42, D49, D56) during the recovery period. Food consumption was measured once or twice weekly over a 24-h period. When food consumption was assayed, 200 g of animal feed was delivered; the remaining portions were measured at the same time on the second day, and the food consumption was calculated. Specifically, the food intake was measured twice weekly during the treatment period (D2, D6, D9, D13, D16, D20, D23, and D27) and then measured weekly (D34, D41, D48, D55) during the recovery period.

### Ophthalmic examination

During adaptation and at the end of treatment period (D26) and recovery (D54) periods, tropicamide was applied to the mydriasis, cornea, conjunctiva, iris, pupil, crystalline lens, anterior/atria chamber, and fundus oculi of all animals, and they were examined using a binocular indirect ophthalmoscope (Cat. #YZ25C, 6-6 VISION TECH Co., Ltd., Suzhou, Jiangsu province, China).

### CBC, biochemical, and coagulation examination

At the end of treatment period (D28) and recovery (D56), aorta blood (6 ml) was collected from all animals after fasting for 12 h.

For the complete blood count assay, 2 ml of blood was anticoagulated by ethylenediamine tetraacetic acid (EDTA). Hematological parameters, including red blood cells (RBC), white blood cells (WBC), hemoglobin (HGB), hematocrit (HCT), platelets (PLT), mean corpuscular volume (MCV), mean corpuscular hemoglobin (MCH), mean corpuscular hemoglobin concentration (MCHC), reticulocyte (RET%, RET#), neutrophils (NEUT%, NEUT#), lymphocytes (LYMPH%, LYMPH#), monocytes (MONO%, MONO#), eosinophils (EOS%, EOS#) and basophils (BASO%, BASO#) were detected by the Automatic Blood Analyzer (XT-2000IV, SYSMEX, Japan).

For biochemical examination, 2 ml of blood was centrifuged (3000 rpm, 10 min, RT), and serum was prepared for analysis. Parameters, including alanine aminotransferase (ALT), aspartate aminotransferase (AST), alkaline phosphatase (ALP), creatine phosphokinase (CK), total bilirubin (TBIL), glucose (GLU), urea (Urea), creatinine (Crea), triglyceride (TG), cholesterol total (TC), troponin (TP), albumin (ALB), globulin (GLOB), sodium (Na^+^), potassium (K^+^), and chloride ion (Cl^−^) and albumin/globulin (A/G), were detected by Automatic Blood Biochemical Analyzer (Hitachi 7180, Hitachi Hi-Tech, Japan).

1.8 ml of blood was anticoagulated by sodium citrate to detect clotting indexes, and the plasma was separated for examination by centrifugation at 3000 rpm for 10 min at room temperature (RT). Blood coagulation indexes, including prothrombin time (PT), activated partial thromboplastin time (APTT), and fibrinogen (FIB), were detected by an automatic Hemagglutination Analyzer (CA-1500, SYSMEX, Japan).

### Urine examination

Urine examinations were conducted once on day 26 and before the end of the recovery period (D54). Urine parameters, including specific gravity (SG), glucose (GLU), protein (PRO), bilirubin (BIL), urobilinogen (URO), pH, ketones (KET), specific gravity (SG), occult blood (BLO), nitrogen (NIT), turbidity (TURB), WBC and pigment, were detected by Urine Analyzer (Zhongke Meiling Cryogenics Co., Ltd., Hefei, Anhui province, China).

### Bone marrow examination

Given that the hematological indexes of the animals indicated normal bone marrow hematopoiesis, a bone marrow smear was not required.

### Necropsy and organ weight

Necropsy was conducted at the end of treatment period (on day 29, twelve rats per group) and the remaining rats were autopsied during the recovery period. Prior to dissection, all rats fasted overnight and were euthanized after intraperitoneal injection of sutai and serrazine hydrochloride mixture. A detailed macroscopic examination was performed on the systemic organs and tissues. The wet weight of the following organs was measured, and the organ coefficients and organ brain coefficients were calculated separately: the brain, spleen, thymus, heart, liver, testis (bilateral)/ovaries (bilateral), epididymis (bilateral), kidneys (bilateral), adrenal glands (bilateral), and uterus (including the cervix) were separated and weighed. For paired organs, the weights of both organs were added together.

### Histopathological examination

The following organs and tissues were extracted from the rats and fixed in 10% neutral buffered formalin: the brain, heart, aorta, pituitary, thyroid, parathyroid, lung including bronchi, liver, trachea, spleen, kidney, adrenal glands, salivary glands (submandibular), esophagus, stomach, duodenum, jejunum, ileum, cecum, colon, rectum, pancreas, epididymis, prostate, seminal vesicle, ovary, uterus (including the cervix), vagina, urinary bladder, submandibular lymph node, mesenteric lymph node, mammary gland (female and male), mammary gland skin, sternum (including bone marrow), femur (including bone marrow), skeletal muscle (biceps flexor cruris), nervi ischiadicus, spinal cord (neck, chest and waist) and abnormal tissues and tissue masses were visually observed. The testes, eyeballs, optic nerve and the harderian gland were fixed with Davidson’s fixative.

All tissues/organs were fixed, and tissue slides were prepared by a standardized process, including dehydration, paraffin embedding, trimming, sectioning by microtome, and staining with hematoxylin-eosin (H&E). A histopathological examination was performed on the toxic target organs of dissected animals. The photomicroscopic assessment and the histopathology slides were viewed at various magnifications (×100 and ×400) to detect pathological lesions using a BX60 photomicroscope (Olympus, Tokyo, Japan). We invited pathologist Yingyong from the Shandong Academy of Pharmaceutical Sciences to peer review the sections.

### Statistical analysis

Data were calculated and analyzed with SPSS (version 20.0, IBM, Armonk, NY). All values were presented as mean ± standard deviation. Quantitative indicators were statistically tested by one-way analysis of variance (ANOVA). Levene’s homogeneity of variance was conducted to assess the homogeneity of variance. When the variance was heterogeneous (*p* ≤ 0.05), the Kruskal–Wallis H rank-sum test (K-W method) was used, When the difference was statistically significant (*p* ≤ 0.05), the Mann-Whitney U rank-sum test (M-W method) was used to compare the group differences. When variance was homogeneous (*p* > 0.05) and one-way analysis of variance (ANOVA) was statistically significant (*p* ≤ 0.05), differences between groups were compared using the Dunnett method.

Comparisons of differences were performed among groups CN, TA, TB, and TC, and all statistical tests were two-sided (*α* = 0.05). Statistical analysis was performed using SPSS, and male and female data calculations and statistical analysis were performed separately. Ophthalmic examination results were not counted and expressed as individual data.

## Results

### Death of animals

There were two deaths of female rats (rat numbers: 4110 and 4111) in group TC (5.0 g/kg/d QZZD) before administration on D15 and D9, with no significant abnormalities in weight, food intake and clinical symptoms. A gross necropsy revealed yellow liquid around the lips and enlarged lungs in both rat. The histopathological report showed mild congestion of the heart, liver, kidney, thyroid, adrenal gland, and ovary, moderate pulmonary congestion, edema, and emphysema in rat 4110, while in rat 4111, there were mild congestion of the heart, liver, kidney, pituitary, and ovary, as well as moderate pulmonary congestion and emphysema.

It was thus concluded that the deaths were attributed to improper administration. No other deaths were observed until the end of the experiment.

### Clinical signs observation

There were no abnormal symptoms in group CN and group TA (0.5 g/kg/d QZZD) during the experiment. In group TB (1.5 g/kg/d QZZD) from D20 to D30, there was blue coloring of part of the tail skin of some rats, and the bedding and feces in the cage also turned blue. In group TC (5.0 g/kg/d QZZD) rats exhibited partial blue staining of the tail skin from D15 to D52, and part of the bedding and feces in the cage turned blue from D16 to D38. After the recovery period, the above symptoms disappeared.

### Body weight and food consumption

**FIGURE 2:**
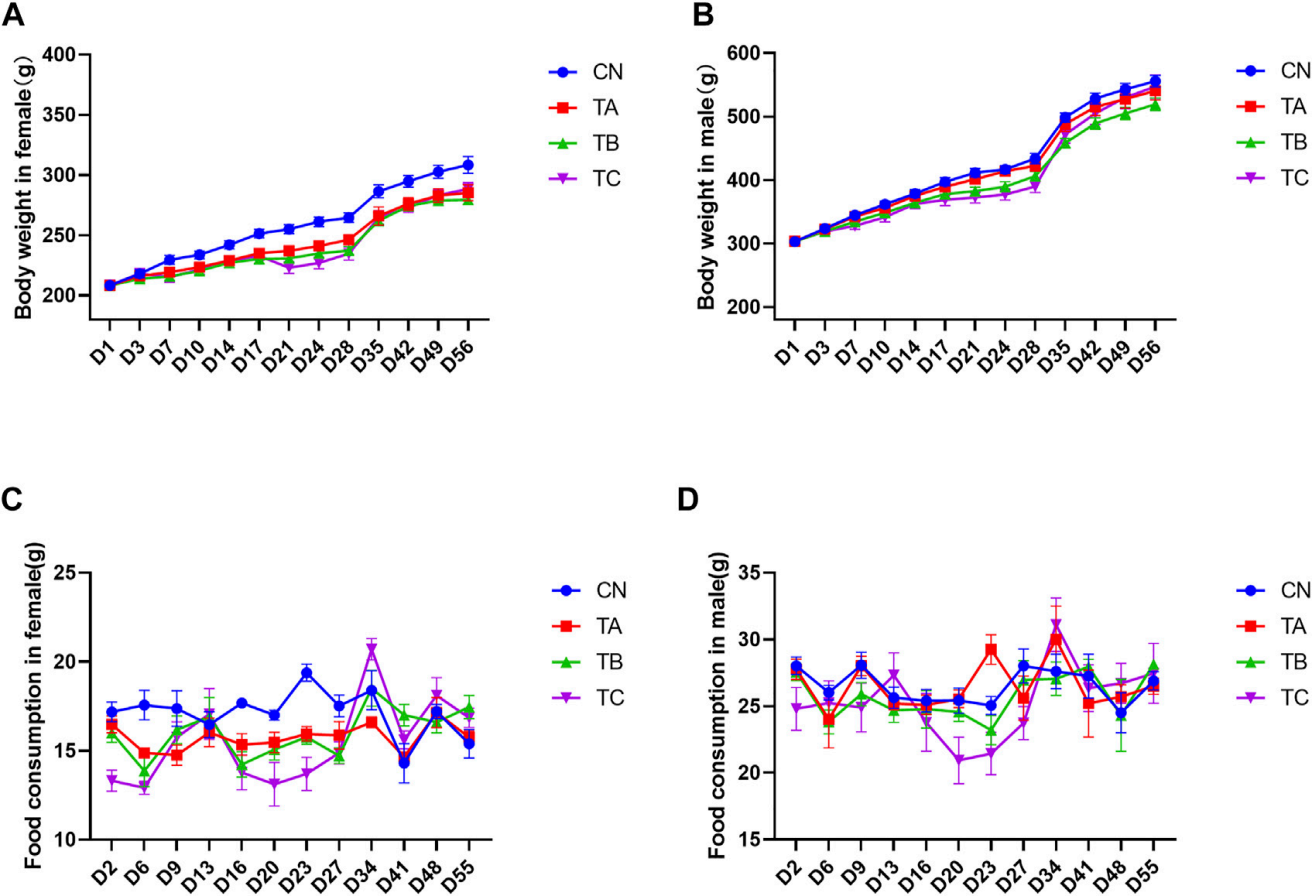
Effects of different doses of QZZD on the body weight in female **(A)** or male **(B)** rats and food consumption in female **(C)** or male **(D)** rats. In the treatment period, *n* = 18 in each group, and two female rats (4110 and 4111) death caused by faulty gavage operation, the total number was 16 (*n* = 16) in TC female group. In the recovery period, *n* = 6 in each group for female or male.

Changes in body weight are widely acknowledged as an important indicator of adverse effects of herbs. During the experiment, there was significant body weight decrease of female rats from D14 to D56 in group TA (0.5 g/kg/d QZZD), while no significant differences were observed in male rats. In group TB (1.5 g/kg/d QZZD), the body weight of female rats was decreased from D7 to D56 and that of male rats was decreased from D21 to D24. In group TC (5.0 g/kg/d QZZD), body weight decrease was observed from D7 to D49 in females and from D17 to D28 in males. During the drug administration period, weight decrease exhibited a good dose-response relationship and time-response relationship. Therefore, it may be attributed to the tested formula. The female rats in group TA (0.5 g/kg/d QZZD) showed smaller body decrease and exhibited normal behavior with no significant organ injury. Detailed analysis is shown in Figures 2A,B.

During drug administration, the average food intake was decreased on D6 and D23 in females (*p* < 0.05) and was increased on D23 in males (*p* < 0.05) in group TA (0.5 g/kg/d QZZD). Group TB (1.5 g/kg/d QZZD) witnessed decrease in females on D6, D16, D23 and D27 (*p* < 0.05), while there was no significant difference in males. Group TC (5.0 g/kg/d QZZD) showed decrease of the average food intake on D2, D6, D16, D20, D23, and D27 in females (*p* < 0.05) and in males on D20 (*p* < 0.05). During the recovery period, the food intake of all groups returned to normal. The decrease of food intake and duration in females of the above groups showed a good dose-response relationship, which was considered to be related to the tested formula. Although fluctuations were observed in few males, the overall food intake remained relatively stable. In group TA (0.5 g/kg/d QZZD), females exhibited less variations in average food intake, the abnormal food intake were scattered in time, and the duration was shorter. It can be concluded that the overall food intake in group TA (0.5 g/kg/d QZZD) was relatively stable and normal. More detailed analysis is shown in Figures 2C,D.

### Ophthalmologic examination

Ophthalmic examination of animals during the experiment showed no significant abnormalities. Individual data are shown in Supplementary Table S3.

### Complete blood count results

Hematological parameters, including the Complete Blood Count, are sensitive indicators of toxicity and serves as an important index of health status for humans and animals (Ding et al., 2021). At the end of administration, there was no significant difference in hematological indexes of female between group TA (0.5 g/kg/d QZZD) and group CN, while the RET% of males in group TA (0.5 g/kg/d QZZD) was significantly increased (*p* < 0.05). In group TB (1.5 g/kg/d QZZD), RET# and RET% were increased in females (*p* < 0.05), while NEUT#, NEUT%, RET# and RET% were increased in males (*p* < 0.05). In group TC (5.0 g/kg/d QZZD), RBC and MCHC were significantly decreased in female rats (*p* < 0.05), while PLT, MCV, MCH, RET#, and RET% were significantly increased (*p* < 0.05); in males, the RBC, HGB, HCT, and EO% were significantly decreased (*p* < 0.05), while WBC, PLT, NEUT#, MONO#, RET# and RET% were increased (*p* < 0.05).

At the end of the recovery period, there were no significant differences in CBC parameters of females between group TA (0.5 g/kg/d QZZD) and group CN, while the RET# and RET% of males in group TA (0.5 g/kg/d QZZD) were significantly decreased (*p* < 0.05). In group TB (1.5 g/kg/d QZZD), there were no significant differences in hematological indexes of females, while the MCHC of males was significantly decreased (*p* < 0.05). In group TC (5.0 g/kg/d QZZD), the MCV of females was significantly increased (*p* < 0.05), while RET# and RET% of males was decreased (*p* < 0.05). The red blood cell count in the recovery stage returned to normal, and no significant changes in RET#, RET%, MCHC and MCV were observed. Detailed analysis is shown in Table 2.

**TABLE 2:** Effect of QZZD on hematological parameters in the sub-chronic oral toxicity study.

Parameter (mean ± SD)	Treatment period (*n* = 12)				Recovery period (*n* = 6)			
	CN	TA	TB	TC	CN	TA	TB	TC
Female								
WBC(10^9^/L)	4.87 ± 1.38	4.61 ± 1.66	6.12 ± 2.04	5.20 ± 1.27	4.53 ± 1.04	4.54 ± 1.29	4.53 ± 1.32	5.40 ± 1.51
RBC(10^12^/L)	7.24 ± 0.22	7.25 ± 0.38	6.95 ± 0.50	6.74 ± 0.50^*^	7.58 ± 0.33	7.83 ± 0.27	7.68 ± 0.27	7.66 ± 0.18
HGB (g/L)	140.33 ± 6.01	140.17 ± 7.00	136.17 ± 7.69	135.50 ± 5.48	146.83 ± 5.23	151.00 ± 5.62	148.67 ± 4.76	151.17 ± 3.71
PLT (10^9^/L)	1164.92 ± 123.32	1171.25 ± 97.27	1181.08 ± 113.10	1505.40 ± 139.32^*^	1105.00 ± 80.38	1008.33 ± 81.16	1075.33 ± 68.60	1002.33 ± 147.20
HCT (%)	36.74 ± 1.40	36.62 ± 1.28	35.89 ± 1.80	36.21 ± 1.45	38.70 ± 1.59	39.93 ± 1.23	40.08 ± 1.24	40.77 ± 1.17
MCV(fL)	50.77 ± 1.61	50.58 ± 1.90	51.75 ± 2.15	53.85 ± 2.59^*^	51.08 ± 1.00	51.00 ± 1.19	52.22 ± 1.86	53.28 ± 1.49^*^
MCH(pg)	19.38 ± 0.50	19.35 ± 0.63	19.62 ± 0.56	20.14 ± 0.86^*^	19.38 ± 0.32	19.28 ± 0.28	19.35 ± 0.46	19.77 ± 0.53
MCHC(g/L)	381.92 ± 6.72	382.83 ± 8.29	379.33 ± 6.41	374.30 ± 6.78^*^	379.50 ± 4.59	378.17 ± 6.31	371.00 ± 9.34	370.67 ± 2.80
NEUT% (%)	17.76 ± 7.12	20.01 ± 8.14	21.23 ± 11.06	23.99 ± 12.02	17.53 ± 3.43	20.43 ± 8.36	15.73 ± 6.03	15.37 ± 4.86
LYMPH% (%)	78.39 ± 7.42	75.81 ± 8.03	75.68 ± 11.37	71.95 ± 12.66	77.42 ± 3.23	74.45 ± 8.32	78.25 ± 6.21	80.53 ± 5.03
MONO% (%)	2.68 ± 1.25	2.86 ± 0.76	2.30 ± 1.05	3.26 ± 1.23	3.72 ± 0.75	3.52 ± 0.55	4.60 ± 4.04	3.33 ± 0.94
EO% (%)	1.18 ± 0.60	1.33 ± 0.64	0.79 ± 0.36	0.80 ± 0.33	1.33 ± 0.24	1.60 ± 0.88	1.42 ± 0.54	0.77 ± 0.28
BASO% (%)	0.00 ± 0.00	0.00 ± 0.00	0.00 ± 0.00	0.00 ± 0.00	0.00 ± 0.00	0.00 ± 0.00	0.00 ± 0.00	0.00 ± 0.00
NEUT#(10^9^/L)	0.83 ± 0.37	0.95 ± 0.67	1.35 ± 0.94	1.21 ± 0.66	0.80 ± 0.25	1.00 ± 0.66	0.70 ± 0.28	0.79 ± 0.23
LYMPH#(10^9^/L)	3.86 ± 1.31	3.47 ± 1.18	4.59 ± 1.55	3.78 ± 1.21	3.51 ± 0.83	3.32 ± 0.69	3.54 ± 1.12	4.39 ± 1.39
MONO#(10^9^/L)	0.13 ± 0.08	0.13 ± 0.06	0.14 ± 0.06	0.17 ± 0.07	0.17 ± 0.04	0.16 ± 0.06	0.22 ± 0.22	0.18 ± 0.08
EO#(10^9^/L)	0.05 ± 0.02	0.06 ± 0.03	0.05 ± 0.02	0.04 ± 0.02	0.06 ± 0.01	0.07 ± 0.03	0.06 ± 0.02	0.04 ± 0.02
BASO#(10^9^/L)	0.00 ± 0.00	0.00 ± 0.00	0.00 ± 0.00	0.00 ± 0.00	0.00 ± 0.00	0.00 ± 0.00	0.00 ± 0.00	0.00 ± 0.00
RET#(10^12^/L)	0.2389 ± 0.0400	0.2670 ± 0.0419	0.3392 ± 0.0657^*^	0.5621 ± 0.1479^*^	0.2068 ± 0.0541	0.2032 ± 0.0242	0.2078 ± 0.0408	0.2156 ± 0.0282
RET%	3.301 ± 0.542	3.693 ± 0.627	4.901 ± 1.020^*^	8.373 ± 2.083^*^	2.715 ± 0.601	2.595 ± 0.300	2.712 ± 0.548	2.823 ± 0.421
Male								
WBC(10^9^/L)	7.41 ± 1.56	8.53 ± 2.92	9.29 ± 1.78	9.97 ± 1.55^*^	7.34 ± 1.54	6.78 ± 1.10	6.63 ± 0.67	6.62 ± 1.25
RBC(10^12^/L)	8.16 ± 0.33	8.09 ± 0.20	7.91 ± 0.49	7.46 ± 0.40^*^	8.38 ± 0.56	8.25 ± 0.18	8.27 ± 0.20	8.38 ± 0.35
HGB (g/L)	155.67 ± 6.17	153.58 ± 4.62	149.08 ± 9.37	139.67 ± 6.83^*^	152.67 ± 7.50	152.67 ± 4.37	153.00 ± 8.32	158.50 ± 4.68
PLT (10^9^/L)	1163.92 ± 96.90	1130.67 ± 139.77	1272.83 ± 187.87	1466.42 ± 118.05^*^	1126.33 ± 167.22	1140.00 ± 98.81	1055.00 ± 123.94	1095.17 ± 68.36
HCT (%)	40.76 ± 1.67	40.29 ± 1.35	39.40 ± 2.31	37.18 ± 1.85^*^	40.20 ± 1.76	40.13 ± 1.26	41.52 ± 2.01	42.28 ± 1.51
MCV(fL)	50.02 ± 1.86	49.84 ± 2.05	49.82 ± 1.54	49.92 ± 2.63	48.10 ± 1.91	48.65 ± 1.43	50.22 ± 2.51	50.50 ± 1.95
MCH(pg)	19.08 ± 0.50	19.00 ± 0.64	18.84 ± 0.42	18.74 ± 0.72	18.25 ± 0.47	18.53 ± 0.31	18.50 ± 0.98	18.95 ± 0.63
MCHC(g/L)	382.00 ± 7.50	381.33 ± 6.49	378.50 ± 10.96	375.83 ± 8.22	379.83 ± 5.81	380.50 ± 8.26	368.33 ± 4.27^*^	374.83 ± 2.71
NEUT% (%)	12.91 ± 3.26	13.46 ± 4.41	20.01 ± 9.35^*^	17.53 ± 6.77	18.60 ± 2.36	16.20 ± 3.45	18.10 ± 5.22	16.80 ± 3.49
LYMPH% (%)	83.46 ± 3.88	82.63 ± 5.05	76.35 ± 10.08	79.23 ± 7.03	74.27 ± 3.53	76.75 ± 3.29	75.48 ± 5.10	76.63 ± 4.66
MONO% (%)	2.68 ± 0.78	3.23 ± 0.89	2.91 ± 1.12	2.75 ± 0.59	5.67 ± 2.04	5.72 ± 0.82	5.22 ± 1.22	5.43 ± 1.30
EO% (%)	0.95 ± 0.42	0.68 ± 0.19	0.73 ± 0.33	0.49 ± 0.21^*^	1.47 ± 0.88	1.33 ± 0.19	1.20 ± 0.33	1.13 ± 0.46
BASO% (%)	0.00 ± 0.00	0.00 ± 0.00	0.00 ± 0.00	0.00 ± 0.00	0.00 ± 0.00	0.00 ± 0.00	0.00 ± 0.00	0.00 ± 0.00
NEUT#(10^9^/L)	0.94 ± 0.26	1.11 ± 0.49	1.91 ± 1.12^*^	1.70 ± 0.61^*^	1.36 ± 0.27	1.08 ± 0.16	1.20 ± 0.37	1.12 ± 0.35
LYMPH#(10^9^/L)	6.21 ± 1.45	7.09 ± 2.65	7.04 ± 1.50	7.95 ± 1.75	5.45 ± 1.21	5.23 ± 1.03	5.00 ± 0.61	5.07 ± 1.00
MONO#(10^9^/L)	0.20 ± 0.06	0.27 ± 0.13	0.27 ± 0.13	0.27 ± 0.05^*^	0.43 ± 0.19	0.39 ± 0.07	0.35 ± 0.10	0.35 ± 0.07
EO#(10^9^/L)	0.07 ± 0.03	0.06 ± 0.02	0.07 ± 0.02	0.05 ± 0.02	0.11 ± 0.07	0.09 ± 0.03	0.08 ± 0.03	0.08 ± 0.03
BASO#(10^9^/L)	0.00 ± 0.00	0.00 ± 0.00	0.00 ± 0.00	0.00 ± 0.00	0.00 ± 0.00	0.00 ± 0.00	0.00 ± 0.00	0.00 ± 0.00
RET#(10^12^/L)	0.2789 ± 0.0356	0.3100 ± 0.0421	0.3863 ± 0.0717^*^	0.5739 ± 0.1246^*^	0.2997 ± 0.0210	0.2617 ± 0.0176^*^	0.2538 ± 0.0630	0.2329 ± 0.0200^*^
RET%	3.413 ± 0.356	3.830 ± 0.482^*^	4.915 ± 1.100^*^	7.685 ± 1.585^*^	3.585 ± 0.243	3.173 ± 0.212^*^	3.070 ± 0.765	2.780 ± 0.211^*^

Compared with control group, *p* ≤ 0.05.

QZZD, 5.0 g/kg group, two female rats (4110 and 4111) death caused by faulty gavage operation in treatment period, resulting in animal sample reduction, the total number was 10 (*n* = 10).

TA, (treatment group A, 0.5 g/kg/day QZZD); TB, (treatment group B, 1.5 g/kg/day QZZD); TC, (treatment group C, 5.0 g/kg/day QZZD); CN, (control group, 20 ml/kg pure water); WBC, white blood cell; RBC, red blood cell; HGB, hemoglobin; PLT, Platelet; HCT%, hematocrit percentage; MCV, mean corpuscular volume; MCH, mean corpuscular hemoglobin; MCHC, mean corpuscular hemoglobin concentration; NEUT%, neutrophil percentage; LYMPH%, lymphocyte percentage; MONO%, monocyte percentage; EO%, eosinophil percentage; BASO%, basophil percentage; NEUT#,neutrophil; LYMPH#, lymphocyte; MONO#, monocyte; EO#, eosinophil; BASO#, basophil; RET #, reticulocytes; RET %, reticulocytes percentage.

### Blood biochemical and coagulation indicators assessment

It is well-acknowledged that serum biochemical parameters and coagulation indexes play an important part during toxicological evaluation. Data of the biochemical parameters in rats at the end of the treatment and recovery periods are shown in Table 3. At the end of the treatment period, there was significant increase of TBIL in both females and males in group TA (0.5 g/kg/d QZZD) compared with group CN (*p* < 0.05). In group TB (1.5 g/kg/d QZZD), there was increase of TBIL in males, while in females there were decrease of TG and increase of TBIL (*p* < 0.05). In group TC (5.0 g/kg/d QZZD), TBIL, Urea, and K+ were significantly increased and CL-was decreased in females, while TBIL and K+ were increased and TG and CL-decreased in males (*p* < 0.05).

**TABLE 3:** Effect of QZZD on biochemical and coagulation parameters in the sub-chronic oral toxicity study.

Parameter (mean ± SD)	Treatment period (*n* = 12)				Recovery period (*n* = 6)			
	CN	TA	TB	TC	CN	TA	TB	TC
Female								
ALT (U/L)	40.48 ± 5.19	40.39 ± 9.83	39.95 ± 8.74	37.50 ± 14.61	44.92 ± 7.39	40.00 ± 6.81	47.78 ± 3.00	41.33 ± 4.50
AST (U/L)	140.59 ± 25.16	116.68 ± 23.99	127.78 ± 33.45	140.86 ± 36.80	139.58 ± 21.98	132.97 ± 31.63	147.72 ± 36.54	117.48 ± 21.48
TP (g/L)	61.08 ± 3.65	62.43 ± 3.11	59.85 ± 2.15	63.39 ± 3.80	68.85 ± 4.09	64.92 ± 2.95	64.20 ± 1.97	67.85 ± 5.95
ALB (g/L)	29.47 ± 1.57	30.44 ± 1.88	29.13 ± 1.48	30.06 ± 1.21	32.85 ± 2.15	30.48 ± 1.95	31.13 ± 1.11	32.45 ± 3.05
TBIL (mmol/L)	1.78 ± 0.36	2.56 ± 0.75^*^	3.84 ± 1.80^*^	7.98 ± 4.20^*^	1.79 ± 0.43	1.39 ± 0.44	1.02 ± 0.31^*^	1.11 ± 0.44^*^
ALP(U/L)	93.00 ± 16.03	85.67 ± 16.39	94.83 ± 17.68	120.30 ± 37.46	72.50 ± 31.28	67.00 ± 11.51	91.33 ± 22.66	83.50 ± 8.73
GLU (mmol/L)	7.68 ± 1.23	7.95 ± 1.33	7.87 ± 1.09	7.86 ± 1.17	8.39 ± 1.73	7.89 ± 1.00	9.33 ± 1.33	10.25 ± 2.66
Urea (mmol/L)	5.85 ± 1.07	5.25 ± 1.06	5.65 ± 0.72	8.63 ± 5.24^*^	6.65 ± 1.50	6.01 ± 1.48	6.55 ± 1.08	7.20 ± 1.37
Crea (mmol/L)	31.47 ± 4.88	31.57 ± 5.57	27.15 ± 3.67	31.25 ± 10.31	34.97 ± 4.11	33.55 ± 3.46	40.93 ± 6.22	29.78 ± 4.27
TC (mmol/L)	1.59 ± 0.29	1.56 ± 0.31	1.50 ± 0.19	1.91 ± 0.53	1.87 ± 0.38	1.68 ± 0.40	1.75 ± 0.35	1.82 ± 0.37
TG (mmol/L)	0.41 ± 0.10	0.44 ± 0.09	0.35 ± 0.13	0.40 ± 0.13	0.74 ± 0.40	0.58 ± 0.18	0.45 ± 0.14	0.56 ± 0.31
CK(U/L)	956.83 ± 311.83	751.58 ± 318.33	931.75 ± 385.25	1146.10 ± 533.25	970.17 ± 199.83	924.00 ± 306.83	960.00 ± 530.28	802.83 ± 160.32
K^+^(mmol/L)	4.54 ± 0.22	4.56 ± 0.27	4.44 ± 0.24	4.87 ± 0.31^*^	4.50 ± 0.18	4.32 ± 0.23	4.55 ± 0.52	4.48 ± 0.24
Na^+^(mmol/L)	141.92 ± 0.90	142.42 ± 1.83	142.08 ± 1.31	141.40 ± 1.51	141.50 ± 1.05	141.83 ± 0.41	141.83 ± 1.94	141.50 ± 0.55
Cl^−^(mmol/L)	106.58 ± 1.51	105.83 ± 1.95	105.50 ± 1.17	102.20 ± 3.16^*^	103.67 ± 1.03	103.33 ± 1.37	102.67 ± 2.07	102.33 ± 1.21
GLOB(g/L)	31.62 ± 2.67	31.98 ± 1.74	30.73 ± 1.61	33.33 ± 2.95	36.00 ± 2.14	34.43 ± 1.76	33.07 ± 2.04	35.40 ± 3.33
A/G	0.94 ± 0.07	0.95 ± 0.06	0.95 ± 0.07	0.91 ± 0.06	0.92 ± 0.04	0.89 ± 0.06	0.95 ± 0.08	0.92 ± 0.06
PT (sec)	15.16 ± 0.37	15.28 ± 0.47	15.65 ± 0.36^*^	16.65 ± 1.06^*^	8.17 ± 0.05	8.22 ± 0.12	8.13 ± 0.31	8.28 ± 0.44
APTT (sec)	11.82 ± 0.93	11.74 ± 0.77	11.74 ± 0.97	13.23 ± 1.47^*^	12.07 ± 1.19	11.17 ± 0.55	11.58 ± 0.97	12.12 ± 0.85
Fbg (g/L)	1.98 ± 0.23	1.93 ± 0.16	1.89 ± 0.14	2.08 ± 0.30	1.74 ± 0.08	1.87 ± 0.20	1.60 ± 0.17	1.70 ± 0.14
Male								
ALT (U/L)	48.56 ± 7.96	41.11 ± 8.49	39.25 ± 7.96	43.44 ± 9.72	46.00 ± 7.56	42.60 ± 11.50	43.57 ± 3.51	43.15 ± 5.17
AST (U/L)	148.18 ± 50.12	129.25 ± 43.52	128.49 ± 42.27	125.30 ± 47.06	132.75 ± 34.89	117.33 ± 21.41	100.07 ± 14.56	123.58 ± 27.49
TP (g/L)	59.88 ± 2.77	59.64 ± 2.89	58.72 ± 3.82	59.12 ± 3.51	63.13 ± 2.78	62.53 ± 1.86	62.55 ± 2.68	62.25 ± 3.31
ALB (g/L)	26.69 ± 0.83	26.98 ± 0.95	27.09 ± 1.21	27.17 ± 1.07	27.63 ± 0.49	27.15 ± 0.94	26.93 ± 1.31	27.83 ± 0.80
TBIL (mmol/L)	1.74 ± 0.26	2.15 ± 0.44^*^	2.29 ± 0.64^*^	7.43 ± 3.01^*^	1.50 ± 0.21	1.47 ± 0.38	1.37 ± 0.38	1.16 ± 0.26
ALP(U/L)	183.25 ± 40.33	178.00 ± 29.01	180.67 ± 46.38	236.17 ± 97.25	133.17 ± 22.24	119.33 ± 31.45	121.33 ± 20.19	145.33 ± 35.03
GLU (mmol/L)	9.03 ± 1.51	8.12 ± 1.48	7.97 ± 1.07	8.91 ± 2.05	8.26 ± 1.08	8.60 ± 1.92	8.58 ± 1.54	9.28 ± 1.54
Urea (mmol/L)	5.67 ± 0.74	4.84 ± 1.07	5.18 ± 0.63	6.38 ± 1.29	5.65 ± 1.04	6.05 ± 1.56	5.66 ± 0.71	5.75 ± 1.08
Crea (mmol/L)	25.41 ± 2.04	24.38 ± 4.43	26.11 ± 7.27	26.02 ± 2.86	25.35 ± 3.22	25.92 ± 3.47	26.22 ± 3.51	24.63 ± 4.29
TC (mmol/L)	1.47 ± 0.29	1.45 ± 0.24	1.31 ± 0.30	1.53 ± 0.41	1.66 ± 0.22	1.58 ± 0.12	1.65 ± 0.23	1.40 ± 0.28
TG (mmol/L)	0.53 ± 0.20	0.42 ± 0.13	0.38 ± 0.11^*^	0.28 ± 0.13^*^	0.83 ± 0.07	0.73 ± 0.31	0.60 ± 0.18	0.77 ± 0.25
CK(U/L)	1161.75 ± 714.29	822.42 ± 640.61	768.33 ± 494.33	909.75 ± 587.29	944.33 ± 518.88	786.83 ± 360.22	555.00 ± 201.42	760.17 ± 347.95
K^+^(mmol/L)	5.00 ± 0.30	5.03 ± 0.23	5.53 ± 0.97	5.39 ± 0.22^*^	4.87 ± 0.30	4.88 ± 0.17	4.77 ± 0.12	4.83 ± 0.28
Na^+^(mmol/L)	143.25 ± 1.22	142.92 ± 1.16	143.25 ± 1.29	143.17 ± 1.34	142.00 ± 0.89	142.00 ± 1.10	143.17 ± 1.17	143.00 ± 0.89
Cl^−^(mmol/L)	105.67 ± 1.50	105.83 ± 1.34	105.33 ± 2.15	103.00 ± 1.54^*^	103.50 ± 1.38	101.83 ± 1.72	103.00 ± 1.67	102.67 ± 1.03
GLOB(g/L)	33.18 ± 2.47	32.66 ± 2.24	31.63 ± 2.92	31.95 ± 2.64	35.50 ± 2.46	35.38 ± 1.53	35.62 ± 2.06	34.42 ± 2.93
A/G	0.81 ± 0.06	0.83 ± 0.05	0.86 ± 0.06	0.85 ± 0.04	0.78 ± 0.05	0.77 ± 0.04	0.76 ± 0.05	0.82 ± 0.06
PT (sec)	15.38 ± 0.45	15.29 ± 0.41	15.75 ± 0.52	17.15 ± 0.77^*^	8.77 ± 0.34	9.47 ± 1.10	9.08 ± 0.66	9.08 ± 0.47
APTT (sec)	13.56 ± 0.72	13.24 ± 1.13	12.24 ± 1.14	13.06 ± 1.63	12.95 ± 0.96	13.40 ± 0.79	14.08 ± 0.88	13.35 ± 1.31
Fbg (g/L)	2.35 ± 0.14	2.36 ± 0.12	2.36 ± 0.18	2.49 ± 0.55	2.01 ± 0.06	2.03 ± 0.07	2.15 ± 0.10^*^	1.97 ± 0.10

Compared with control group, *p* ≤ 0.05.

QZZD, 5.0 g/kg group, two female rats (4110 and 4111) death caused by faulty gavage operation in treatment period, resulting in animal sample reduction, the total number was 10 (*n* = 10).

TA, (treatment group A, 0.5 g/kg/day QZZD); TB, (treatment group B, 1.5 g/kg/day QZZD); TC, (treatment group C, 5.0 g/kg/day QZZD); CN, (control group, 20 ml/kg pure water); ALT, alanine aminotransferase; AST, aspartate aminotransferase; TP, total protein; ALB, albumin; TBIL, total bilirubin; ALP, alkaline phosphatase; GLU, glucose; Crea, Creatinine; TC, total cholesterol; TG, triglyceride; CK, creatine kinase; K, potassium; Na, Sodium; Cl, Chloride; GLOB, Globulin; A/G,Albumin/globulin; PT, Partial Thromboplastin time; APTT, Activated partial thromboplastin time; Fbg, Fasting blood glucose.

At the end of the recovery period, TBIL of female rats in group TB (1.5 g/kg/d QZZD) and group TC (5.0 g/kg/d QZZD) was decreased compared with that of group CN. However, TBIL in the two groups were within the normal range. Besides, there were no other significant differences of serum biochemical indexes between males and females in all groups except for those mentioned above.

At the end of treatment, there was no significant difference between group TA (0.5 g/kg/d QZZD) and group CN in both genders. In group TB (1.5 g/kg/d QZZD), PT was significantly increased in females (*p* < 0.05), but there was no significant difference in males. In group TC (5.0 g/kg/d QZZD), there was increase of PT and APTT in females (*p* < 0.05), and increase of PT in males (*p* < 0.05). The increase of PT and APTT mentioned above were within the normal range and were not considered to be related to the formula.

At the end of the recovery period, there was a slight increase of Fbg of male rats in group TB (1.5 g/kg/d QZZD) compared with group CN, which was within the normal range (*p* < 0.05). Given that no significant dose-response relationship was observed, it was not considered related to the tested formula. In addition, all groups exhibited no significant differences in coagulation indexes. Detailed analysis is shown in Table 3.

### Urine examination

It is widely acknowledged that urinary indices can reflect the metabolic status of the body (Lu et al., 2014). As shown in Table 4, all urinary parameters from each group were within the normal physiological range throughout the experimental period, and no significant abnormal changes associated with the tested formula were observed.

**TABLE 4:** Effect of QZZD on urine parameters in the sub-chronic oral toxicity study. (results were presented as number).

Index (mean ± SD)	Treatment period (*n* = 12)				Recovery period (*n* = 6)			
	CN	TA	TB	TC	CN	TA	TB	TC
**Female**								
Appearance	**Colorless/Light Yellow/Yellow (n/n)**							
	1/11/0	0/10/2	0/10/2	1/7/2	0/5/1	0/2/4	0/5/1	0/4/2
PH	7.58 ± 0.46	7.5 ± 0.52	7.62 ± 0.31	7.25 ± 0.54	8.25 ± 0.27	8.16 ± 0.4	8.25 ± 0.41	8 ± 0.44
S.G.	1.011 ± 0.002	1.009 ± 0.001	1.01 ± 0.004	1.012 ± 0.004	1.012 ± 0.002	1.013 ± 0.002	1.012 ± 0.002	1.014 ± 0.002
GLU	**0/2.8 (mmol/L,n/n)**							
	12/0	12/0	12/0	10/0	5/1	6/0	6/0	6/0
PRO	**0/0.1/0.2/0.3/0.5 (g/L,n/n)**							
	12/0/0/0/0	12/0/0/0/0	12/0/0/0/0	10/0/0/0/0	5/1/0/0/0	3/2/1/0/0	6/0/0/0/0	3/2/1/0/0
BIL	**Negative/Positive (n/n)**							
	12/0	12/0	12/0	10/0	6/0	6/0	6/0	6/0
URO	**Negative/Positive(n/n)**							
	12/0	12/0	12/0	10/0	6/0	6/0	6/0	6/0
BLD	**Negative/Positive (n/n)**							
	9/3	9/3	11/1	10/0	6/0	6/0	6/0	6/0
KET	**Negative/Positive (n/n)**							
	12/0	12/0	12/0	10/0	6/0	6/0	6/0	6/0
NIT	**Negative/Positive(n/n)**							
	4/8	3/9	5/7	10/0	4/2	3/3	3/3	4/2
LEU	**0/25/75(Leu/ul,n/n)**							
	12/0/0	12/0/0	11/1/0	7/2/1	6/0	6/0	6/0	6/0
TURB	**Negative/Positive(n/n)**							
	11/1	11/1	11/1	10/0	0/6	0/6	0/6	0/6
**Male**								
Appearance	**Colorless/Light Yellow/Yellow (n/n)**							
	1/8/3	0/10/2	0/11/1	0/9/3	0/2/4	0/2/4	0/3/3	0/5/1
PH	7.91 ± 0.28	8 ± 0.21	7.87 ± 0.22	7.81 ± 0.4	8.33 ± 0.25	8.33 ± 0.25	8.25 ± 0.27	8.5 ± 0
S.G.	1.012 ± 0.003	1.014 ± 0.007	1.011 ± 0.002	1.013 ± 0.004	1.012 ± 0.002	1.013 ± 0.002	1.013 ± 0.002	1.011 ± 0.001
GLU	**0/2.8 (mmol/L,n/n)**							
	12/0	12/0	12/0	12/0	6/0	6/0	5/1	6/0
PRO	**0/0.1/0.2/0.3/0.5 (g/L,n/n)**							
	7/3/1/1/0	5/4/2/0/1	9/2/1/0/0	10/1/1/0/0	0/3/2/1/0	2/0/4/0/0	0/3/2/1/0	
BIL	**Negative/Positive (n/n)**							
	12/0	12/0	12/0	12/0	6/0	6/0	6/0	6/0
URO	**Negative/Positive (n/n)**							
	12/0	12/0	12/0	12/0	6/0	6/0	6/0	6/0
BLD	**Negative/Positive (n/n)**							
	12/0	11/1	12/0	10/2	5/1	6/0	6/0	6/0
KET	**Negative/Positive (n/n)**							
	11/1	10/2	12/0	12/0	6/0	5/1	6/0	6/0
NIT	**Negative/Positive (n/n)**							
	4/8	7/5	11/1	11/1	3/3	5/1	2/4	4/2
LEU	**0/25/75 (Leu/ul,n/n)**							
	9/3/0	8/4/0	11/1/0	3/8/1	6/0	6/0	5/1	6/0
TURB	**Negative/Positive (n/n)**							
	10/2	6/6	9/3	10/2	0/6	0/6	0/6	0/6

QZZD, 5.0 g/kg group, two female rats (4110 and 4111) death caused by faulty gavage operation in treatment period, resulting in animal sample reduction, the total number was 10 (*n* = 10).

TA, (treatment group A, 0.5 g/kg/day QZZD); TB, (treatment group B, 1.5 g/kg/day QZZD); TC, (treatment group C, 5.0 g/kg/day QZZD); CN, (control group, 20 ml/kg pure water); PH, potential of hydrogen; S.G., specific gravity; GLU, glucose; PRO, protein; BIL, bilirubin; URO, urbilinogen; BLD, blood; KET, ketone; NIT, nitrite; LEU, leucocyte; TURB, turbidity.

### Organ weights, organ coefficients and organ/brain coefficients

The organ-to-body weight ratio (organ coefficients) is another sensitive index for toxicity evaluation. Generally, changes in organ coefficient occur before morphological changes (Piao et al., 2013). At the end of the treatment period, there was no significant change of the weight and organ coefficients of female rats, while the organ-body ratio of spleen in male rats was increased in group TA (0.5 g/kg/d QZZD) compared with group CN (*p* < 0.05). In group TB (1.5 g/kg/d QZZD), the wet weight of the thymus was decreased, the organ-body ratio of liver, kidney and brain was increased, and the organ-brain ratio of the liver was increased in females (*p* < 0.05); while in males, the organ-body ratio of liver, kidney, spleen and brain was increased, and the organ-brain ratio of spleen was increased (*p* < 0.05). For females in group TC (5.0 g/kg/d QZZD), the wet weight of the heart and thymus was decreased (*p* < 0.05) while that of the liver was increased (*p* < 0.05); the organ-body ratio of liver, spleen, kidney, brain and adrenal gland was increased (*p* < 0.05), the organ-brain ratio of liver and kidney was increased (*p* < 0.05), and the organ-brain ratio of thymus was decreased (*p* < 0.05), For males in group TC (5.0 g/kg/d QZZD), the wet weight of the heart was decreased (*p* < 0.05), and the wet weight of liver and spleen was increased (*p* < 0.05); there was also an increase in organ-body ratio of liver, spleen, kidney and brain, and in organ-brain ratio of liver and spleen (*p* < 0.05), while the organ-brain ratio of heart was decreased (*p* < 0.05).

**FIGURE 3:**
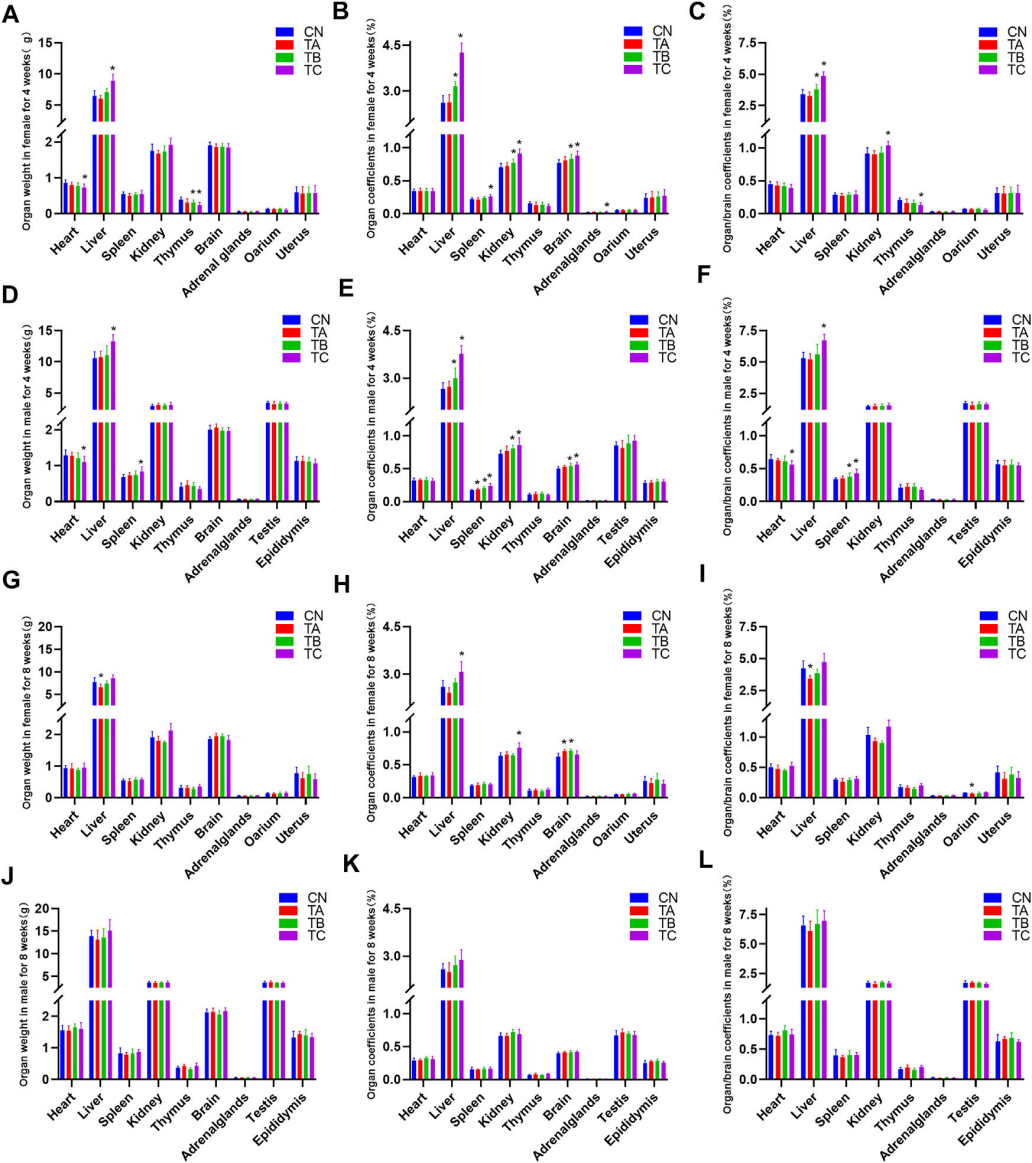
Effects of three different doses of QZZD on absolute organ weights, organ coefficients and organ/brain coefficients in male or female rats at the end of the treatment period or recovery period. In the treatment period, *n* = 12 in female group (*n* = 10 in TC) **(A**–**C)**, and *n* = 12 in male group **(D**–**F)**. In the recovery period, *n* = 6 in each group, including six females **(G**–**I)** and six males **(J**–**L)**. Compare with the control group *p < 0.05.

In group TA (0.5 g/kg/d QZZD), there was a decrease in the wet weight and organ-brain ratio of liver and an increase in the organ-body ratio of brain, and the ovary/brain coefficient was decreased at the end of the recovery period (*p* < 0.05). The organ-body ratio of brain in female rats of group TB (1.5 g/kg/d QZZD) was increased (*p* < 0.05). Moreover, the organ coefficients of liver and kidney of females in group TC (5.0 g/kg/d QZZD) was increased (*p* < 0.05). There were no significant differences in weight and organ coefficient of males in each group. Detailed analysis is shown in Figure 3.

### Pathological examination

Macroscopic observation of morphological characteristics and histopathological examination were performed on tissues/organs at the end of the treatment and recovery period, respectively. The macroscopic examination revealed darkening of livers and kidneys in group TB (1.5 g/kg/d QZZD) and group TC (5.0 g/kg/d QZZD) after the treatment period. At the end of the recovery period, the liver and kidneys of all the rats in group TC (5.0 g/kg/d QZZD) turned black. The pathological examination showed normal staining of liver and kidney tissues with no pigmentation or other organic changes. Individual data of macroscopic morphological observation are shown in Supplementary Table S4.

**FIGURE 4:**
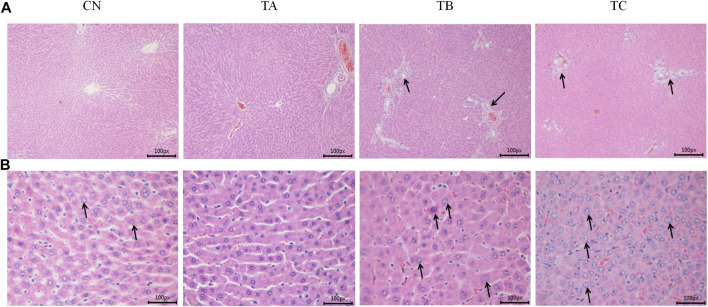
The representative histopathological sections of rat liver at the end of the treatment period. **(A)** The CN and TA rats liver showing normal hepatocytes and hepatic vasculature; The TB and TC rats liverr showing bile duct proliferation and karyomegaly of hepatocytes (×100 magnification). **(B)** The CN and TA rats liver showing normal hepatocytes and hepatic vasculature; The TB and TC rats liver showing bile duct proliferation and karyomegaly of hepatocytes (×400 magnification).

In group TB (1.5 g/kg/d QZZD), histopathological examination at the end of the treatment period exhibited minimal biliary hyperplasia (*n* = 5) and minimal karyomegaly of hepatocytes (*n* = 5). In group TC (5.0 g/kg/d QZZD), there were mild-to-moderate biliary hyperplasia (*n* = 11), and the severity of karyomegaly of hepatocytes ranged from minimal to mild (*n* = 11). After 28 days of drug withdrawal, related lesions recovered completely. The representative histopathological changes after treatment period are shown in Figure 4.

Furthermore, in the experiment period, tissues/organs of SD rats exhibited pathological changes, including basophilic tubules and interstitial mononuclear cell infiltration in the kidney, myocardial cell necrosis and mononuclear cell infiltration in cardiac tissue. These were considered background lesions of SD rats due to their low incidence, mild degree severity and absence of a significant dose-response relationship.

## Discussion

Over the years, the use of herbal medicines has become popular around the world (Wu et al., 2018). The efficacy of herbal medicines is well-established in treating various diseases, being used in traditional and non-traditional forms of medicine for thousands of years (Wang et al., 2019). Meanwhile, much emphasis has been placed on the safety profile of herbal medicine in recent years. Indeed, the low safety profile accounts for the rejection of compounds with druggable profiles in early clinical trials. QZZD is derived from a traditional Chinese medicinal formula, Angong Niuhuang Wan; the efficiency of QZZD has been demonstrated in rat models of VD (Liu et al., 2022). However, the administered doses were not high enough to ensure the clinical safety of QZZD. Therefore, a systematic toxicology study of QZZD in animals is essential to ensure its safety as a therapeutic agent for VD prior to progressing to clinical translation.

It is widely thought that QZZD, composed of *Radix Scutellariae*, *Fructus Gardeniae*, and *Pulvis Fellis Suis*, has heat-clearing and detoxification effects. Current evidence suggests that these components are safe and non-toxic. A toxicity study by Jing et al. found that geniposide did not influence animal mortality, the general condition of the animals, body weights or food consumption. After 4 weeks of treatment, no significant toxicity was observed (Tian et al., 2018). Another study showed that *Radix Scutellariae* exerted no significant adverse effects on the clinical signs, hematology, biochemistry, necropsy, organ weight, and histopathology throughout subacute toxicity tests in rats (Wang et al., 2017). These are consistent with our experimental results.

During the 28-day repeated oral dose toxicity study, QZZD at doses of 0.5, 1.5, and 5.0 g/kg/day caused no death and no treatment-related adverse effects on food consumption, organ weight, ophthalmologic, urinalysis, and hematological parameters. Two female rats in group TC (5.0 g/kg/d QZZD) died during the experiment. Based on our macroscopic and microscopic examination findings, we speculate that the direct cause of death was the incorrectly oral gavage procedure. During the 28-day repeated oral dose toxicity study, QZZD at doses of 0.5, 1.5, and 5.0 g/kg/day caused no death and no treatment-related adverse effects on food consumption, organ weight, ophthalmologic, urinalysis, and hematological parameters. Importantly, some treatment-related changes, including clinical signs (blue-colored stool and tail), body weight (weight loss), serum biochemistry (decreased TG level and increased TBIL level), necropsy findings (liver and kidneys turn black), and histopathological findings (liver bile duct hyperplasia and hepatocyte nuclei hypertrophy), were observed. However, these changes were resolved during the recovery period.

The clinical symptoms of animals is an important indicator to measure the toxicity of the tested drug (Hardisty et al., 2020). During the experiment, the tail skin of rats from the medium and high-dose group turned blue and the bluish coloration of the bedding and feces exhibited a dose-response relationship. The liver and kidney of the medium and high-dose groups turned black, and the clinical signs and macroscopic observations showed good physiological consistency, and these abnormalities disappeared at the end of the recovery period. These abnormalities may be attributed to the pigments in QZZD or its metabolites (Tang et al., 2020).

Body weight and food consumption are used to evaluate the adverse effects caused by the tested drug. And body weight and food consumption are usually correlated and need comprehensive analysis (Dziwenka et al., 2020). Compared with the control group, the female body weight and food consumption of the medium and high dose group was reduced, which had a good dose correlation. In addition, the female rats show variations in body weight and food consumption as compared to male rats. These may be due to high dose and administration volume will contribute to the decrease of gastric volume of rats. The female rats have lower weight and smaller gastric volume compared with the male rats, which predisposes them to gastric volume decrease. Therefore, the food consumption and weight increase in female rats was smaller than that in male rats. In terms of safety evaluation, the fluctuations of body weight and food consumption are small, which fall into the category of physiological fluctuations.

Hematological tests are commonly used to observe the treatment effect and determine whether treatment should be continued or not (Guo et al., 2022). WBC, RBC, HGB, and PLT are the most sensitive indicators in CBC, which are sensitive in response to pathological changes (Mukinda and Eagles, 2010). In the present study, the main indicators RBC, HGB and HCT were moderately reduced in group TC (5.0 g/kg/d QZZD) but remained within the normal physiological reference range; a certain dose-response relationship was observed. Accordingly, these changes were related to the test drug. The increase in RET# and RET% formed part of the adaptive compensatory reaction caused by decreased RBC. The PLT of male and female rats in group TC (5.0 g/kg/d QZZD) was significantly increased, and the coagulation index PT of the animals in the group was increased. Combined with the results of coagulation function, it was believed that the increase in PLT was a compensatory reaction, not secondary to the direct toxicity of the test drug. Combined with the statistical results and individual animal data, it was found that WBC and NEUT# in male animals of the medium- and high-dose groups exhibited an increasing trend, which was believed to be correlated with the test drug. However, the WBC, NEUT#, NEUT%, MONO# and EO% mentioned above were all variations of leukocytes and their classification indexes, which fluctuated slightly within the normal physiological reference range, and the composition proportion of each classification index of leukocyte was also at the normal physiological level. Therefore, they were attributed to physiological fluctuations instead of toxicity-induced injury.

The serum clinical chemistry analysis is highly relevant for the determination of target organs of toxicologic pathology (Weingand et al., 1996). And biochemical indicators are important to evaluate tested drugs for its toxic effect on the liver and kidney functions (Travlos et al., 1996). The TBIL index increased in all treatment groups mentioned above, and a significant increase was observed in group TC (5.0 g/kg/d QZZD), indicating a significant dose-response relationship, which was believed to be related to the test drug. The decrease of TG in medium-dose and high-dose groups male rats exhibited a certain dose correlation, which was thought to be related to the test drug. The variations for K^+^ and CL^−^ indexes were small and fluctuated within the normal physiological reference range, which was not considered abnormal toxicity changes. The increase in Urea levels was caused by exaggerated individual values and was not considered toxicity-induced.

On the organ weights, there was a good dose correlation between the wet weight of the liver, the organ coefficients and organ/brain coefficients at the end of drug administration. Combined with the histological results, it was believed that the variation in the above indexes corresponded to the liver’s adaptive changes during the test drug’s continuous metabolism *in vivo*. The change in other organ indexes was non-significant, and no obvious histopathological abnormalities were found, indicating physiological changes and no obvious toxicity. At the end of the recovery period, the liver wet weight and organ-brain ratio decreased in group TA (0.5 g/kg/d QZZD), although the reduction was not significant, with no histopathological abnormality and no significant dose correlation, which were not attributed to the test drug. Abnormal values were observed for the organ/body ratio or organ/brain ratio, although the difference was not statistically significant and could not be attributed to the test drug.

Anatomical examination and histopathological findings are usually the main sources of toxicities. Observable lesions in the gross anatomy and histopathological examination results might help to determine the possible target organs of toxicities (Olayode et al., 2020). Our study found that QZZD at doses of 1.5 g/kg/d and 5.0 g/kg/d caused bile duct hyperplasia and karyomegaly of hepatocytes in SD rats in the drug treatment group, and the incidence and severity of the lesions were significantly dose-related. Accordingly, the liver lesions in experimental animals were related to drug-reduced. Importantly, these liver lesions fully recovered after 4 weeks of drug withdrawal. The bile duct hyperplasia and karyomegaly of hepatocytes were not seen in recovery groups at the end of the 28-day recovery period indicating these liver lesions were reversible.

Taken together, the repeated administration of QZZD did not cause any toxic effects in rats at doses of up to 0.5 g/kg/d, which corresponds to approximately 16.7 times the proposed dose administered in the clinical trial. The findings of this study substantiate that QZZD has a good safety profile, suitable for the long-term treatment of dementia. And further provide reliable experimental data for rational and safe clinical application of QZZD preparations.

## Conclusion

In conclusion, the present toxicity studies clearly suggest the QZZD has a broad range of safety profiles. QZZD has no systemic toxicity through oral administration. The oral no-observed-adverse-effect-level of QZZD from the 28-day repeated oral dose toxicity study is 0.5 g/kg/d, and no target organ toxicity was identified. Together, the observations from the present study raise no toxicity concern and affirm a broad-spectrum safety of QZZD.

## Data Availability

The original contributions presented in the study are included in the article/Supplementary Material, further inquiries can be directed to the corresponding authors.

## References

[B1] Alam A. Ferdosh S. Ghafoor K. Hakim A. Juraimi A. S. Khatib A. (2016). Clinacanthus nutans: a review of the medicinal uses, pharmacology and phytochemistry. Asian Pac. J. Trop. Med. 9 (4), 402–409. 10.1016/j.apjtm.2016.03.011 27086161

[B2] Chen L. Li M. Yang Z. Tao W. Wang P. Tian X. (2020). Gardenia jasminoides Ellis: ethnopharmacology, phytochemistry, and pharmacological and industrial applications of an important traditional Chinese medicine. J. Ethnopharmacol. 257, 112829. 10.1016/j.jep.2020.112829 32311486

[B3] Chen X. L. Su S. L. Liu R. Qian D. W. Chen L. L. Qiu L. P. (2021). Chemical constituents and pharmacological action of bile acids from animal: a review. Zhongguo Zhong Yao Za Zhi 46 (19), 4898–4906. 10.19540/j.cnki.cjcmm.20210630.201 34738383

[B4] Ding J. Gao X. Zhang F. Zhou Y. Li S. Lu Y. (2021). Toxicological safety evaluation of Qiguiyin formula in rats at the treatment phase and recovery phase. J. Ethnopharmacol. 279, 114364. 10.1016/j.jep.2021.114364 34175446

[B5] Dziwenka M. Coppock R. Alexander M. Palumbo E. Ramirez C. Lermer S. (2020). Safety assessment of a hemp extract using genotoxicity and oral repeat-dose toxicity studies in sprague-dawley rats. Toxicol. Rep. 7, 376–385. 10.1016/j.toxrep.2020.02.014 32123668 PMC7036713

[B6] Gaire B. P. Moon S. K. Kim H. (2014). Scutellaria baicalensis in stroke management: nature's blessing in traditional eastern medicine. Chin. J. Integr. Med. 20 (9), 712–720. 10.1007/s11655-014-1347-9 24752475

[B7] Gao S. M. Liu J. S. Wang M. Cao T. T. Qi Y. D. Zhang B. G. (2018). Traditional uses, phytochemistry, pharmacology and toxicology of codonopsis: a review. J. Ethnopharmacol. 219, 50–70. 10.1016/j.jep.2018.02.039 29501674

[B8] Gua H. Hang L. Wang Q. (2014). Interpretations on the content of the guidance for repetitive dose toxicity testing. Chin. J. Clin. Pharmacol. 30 (08), 744–745. 10.13699/j.cnki.1001-6821.2014.08.028

[B9] Guo X. Weng L. Yi L. Geng D. (2022). Toxicological safety evaluation in acute and 21-day studies of ethanol extract from solanum lyratum thunb. Evid. Based. Complement. Altern. Med. 2022, 8518324. 10.1155/2022/8518324 PMC8991412 35399634

[B10] Hardisty J. F. Harris S. B. Hayes W. A. Weber K. (2020). Oral repeated-dose toxicity studies of BIA 10-2474 in beagle dogs. Regul. Toxicol. Pharmacol. 111, 104555. 10.1016/j.yrtph.2019.104555 31874201

[B11] He J. Liang J. Zhu S. Li J. Zhang Y. Sun W. (2011). Anti-inflammatory effects of Pulvis Fellis Suis extract in mice with ulcerative colitis. J. Ethnopharmacol. 138 (1), 53–59. 10.1016/j.jep.2011.08.019 21872653

[B12] He J. Lv L. Wang Z. Huo C. Zheng Z. Yin B. (2017). Pulvis Fellis Suis extract attenuates ovalbumin-induced airway inflammation in murine model of asthma. J. Ethnopharmacol. 207, 34–41. 10.1016/j.jep.2017.06.016 28624362

[B13] Li C. Wang X. Yan J. Cheng F. Ma X. Chen C. (2020). Cholic acid protects *in vitro* neurovascular units against oxygen and glucose deprivation-induced injury through the BDNF-TrkB signaling pathway. Oxid. Med. Cell. Longev. 2020, 1201624. 10.1155/2020/1201624 33101581 PMC7576336

[B14] Liu S. Cheng F. Ren B. Xu W. Chen C. Ma C. (2022). Qinzhi Zhudan formula improves memory and alleviates neuroinflammation in vascular dementia rats partly by inhibiting the TNFR1-mediated TNF pathway. J. Traditional Chin. Med. Sci. 9, 298–310. 10.1016/j.jtcms.2022.06.011

[B15] Lu Y. Hajifathalian K. Ezzati M. Woodward M. Rimm E. B. Danaei G. (2014). Metabolic mediators of the effects of body-mass index, overweight, and obesity on coronary heart disease and stroke: a pooled analysis of 97 prospective cohorts with 1·8 million participants. Lancet 383 (9921), 970–983. 10.1016/s0140-6736(13)61836-x 24269108 PMC3959199

[B16] Ma C. Wang X. Xu T. Zhang S. Liu S. Zhai C. (2020). An integrative pharmacology-based analysis of refined qingkailing injection against cerebral ischemic stroke: a novel combination of baicalin, geniposide, cholic acid, and hyodeoxycholic acid. Front. Pharmacol. 11, 519. 10.3389/fphar.2020.00519 32457601 PMC7227481

[B17] Mukinda J. T. Eagles P. F. (2010). Acute and sub-chronic oral toxicity profiles of the aqueous extract of Polygala fruticosa in female mice and rats. J. Ethnopharmacol. 128 (1), 236–240. 10.1016/j.jep.2010.01.022 20079821

[B18] Olayode O. A. Daniyan M. O. Olayiwola G. (2020). Biochemical, hematological and histopathological evaluation of the toxicity potential of the leaf extract of Stachytarpheta cayennensis in rats. J. Tradit. Complement. Med. 10 (6), 544–554. 10.1016/j.jtcme.2019.05.001 33134130 PMC7588336

[B19] Piao Y. Liu Y. Xie X. (2013). Change trends of organ weight background data in sprague dawley rats at different ages. J. Toxicol. Pathol. 26 (1), 29–34. 10.1293/tox.26.29 23723565 PMC3620211

[B20] Qi H. Liu R. Zheng W. Zhang L. Ungvari G. S. Ng C. H. (2020). Efficacy and safety of traditional Chinese medicine for tourette's syndrome: a meta-analysis of randomized controlled trials. Asian J. Psychiatr. 47, 101853. 10.1016/j.ajp.2019.101853 31731142

[B21] Rui W. Li S. Xiao H. Xiao M. Shi J. (2020). Baicalein attenuates neuroinflammation by inhibiting NLRP3/caspase-1/GSDMD pathway in MPTP induced mice model of Parkinson's disease. Int. J. Neuropsychopharmacol. 23 (11), 762–773. 10.1093/ijnp/pyaa060 PMC7745250 32761175

[B22] Shi Y. Wei F. Ma S. (2018). Medicinal research progress on pig bile and overview of its quality control. China J. Chin. Materia Medica 43 (04), 637–644. 10.19540/j.cnki.cjcmm.20180104.012 29600634

[B23] Tang X. Wang Y. Yang W. Zheng Y. Liu C. Qu M. (2020). Acute and subchronic oral toxicity study of gardenia yellow E500 in sprague-dawley rats. Int. J. Environ. Res. Public Health 17 (2), E531. 10.3390/ijerph17020531 31947699 PMC7014442

[B24] Tian J. Yi Y. Zhao Y. Li C. Zhang Y. Wang L. (2018). Oral chronic toxicity study of geniposide in rats. J. Ethnopharmacol. 213, 166–175. 10.1016/j.jep.2017.11.008 29128573

[B25] Travlos G. S. Morris R. W. Elwell M. R. Duke A. Rosenblum S. Thompson M. B. (1996). Frequency and relationships of clinical chemistry and liver and kidney histopathology findings in 13-week toxicity studies in rats. Toxicology 107 (1), 17–29. 10.1016/0300-483x(95)03197-n 8597028

[B26] Wang J. Wang L. Lou G. H. Zeng H. R. Hu J. Huang Q. W. (2019). Coptidis rhizoma: a comprehensive review of its traditional uses, botany, phytochemistry, pharmacology and toxicology. Pharm. Biol. 57 (1), 193–225. 10.1080/13880209.2019.1577466 30963783 PMC6461078

[B27] Wang L. Liu Y. Chai J. Du T. Hu J. Zhu R. (2017). Acute and subacute toxicity trials of tetraploid skullcap D20. J. Traditional Chin. Veterinary Med. 36 (03), 11–14. 10.13823/j.cnki.jtcvm.2017.03.003

[B28] Weingand K. Brown G. Hall R. Davies D. Gossett K. Neptun D. (1996). Harmonization of animal clinical pathology testing in toxicity and safety studies. Toxicol. Sci. 29 (2), 198–201. 10.1093/toxsci/29.2.198 8742316

[B29] Wu R. Xiao Z. Zhang X. Liu F. Zhou W. Zhang Y. (2018). The cytochrome P450-mediated metabolism alternation of four effective lignans from schisandra chinensis in carbon tetrachloride-intoxicated rats and patients with advanced hepatocellular carcinoma. Front. Pharmacol. 9, 229. 10.3389/fphar.2018.00229 29593545 PMC5861220

[B30] Xu T. Wang X. Ma C. Ji J. Xu W. Shao Q. (2022). Identification of potential regulating effect of baicalin on NFκB/CCL2/CCR2 signaling pathway in rats with cerebral ischemia by antibody-based array and bioinformatics analysis. J. Ethnopharmacol. 284, 114773. 10.1016/j.jep.2021.114773 34699947

[B31] Zhang H. Lai Q. Li Y. Liu Y. Yang M. (2017). Learning and memory improvement and neuroprotection of Gardenia jasminoides (Fructus gardenia) extract on ischemic brain injury rats. J. Ethnopharmacol. 196, 225–235. 10.1016/j.jep.2016.11.042 27940085

