# Supplementary material for: Toxicological safety evaluation of Qin-Zhi-Zhu-Dan formula in rats during the treatment and recovery periods
Source: Front Pharmacol. 2022 Aug 25;13:987997. doi: 10.3389/fphar.2022.987997 (PMC9453232; doi:10.3389/fphar.2022.987997)
Supplement: Supplementary file 3 [file DataSheet1.PDF]

Table S1. Tentative identification of the main peaks in QZZD in negative mode

Name	Formula	Class	t/min
D-Galactose	C6H12O6	Carbohydrates and derivatives	4.24
Daidzein	C15H10O4	Flavonoids	16.06
Hinokiflavone	C30H18O10	Flavonoids	10.35
Kaempferide	C16H12O6	Flavonoids	14.51
Myricitrin	C21H20O12	Flavonoids	6.02
Oroxin B	C27H30O15	Flavonoids	6.16
p-Hydroxybenzaldehyde	C7H6O2	Phenols	3.46
Pyrogallol	C6H6O3	Phenols	26.96
Steviol-19-O-glucoside	C26H40O8	Terpenoids	10.35
Taxifolin	C15H12O7	Flavonoids	4.29
Tsugaric acid A	C32H50O4	Terpenoids	19.71
Wogonin	C16H12O5	Flavonoids	26.81
Hydroxygenkwanin	C16H12O6	Flavonoids	17.11
Chrysoeriol	C16H12O6	Flavonoids	10.2
Hispidulin	C16H12O6	Flavonoids	18.23
Genistein	C15H10O5	Flavonoids	16.93
apigenin-7-O-glucuronide	C21H18O11	Flavonoids	11.74
Cholic acid	C24H40O5	Terpenoids	10.06
Diosmetin	C16H12O6	Flavonoids	13.74
Iristectorigenin B	C17H14O7	Flavonoids	16.93
Asiatic acid	C30H48O5	Terpenoids	11.34
Baicalein	C15H10O5	Flavonoids	16.5
Betulinic acid	C30H48O3	Terpenoids	15.23
Palmitic Acid	C16H32O2	Fatty Acyls	16.94
Hyodeoxycholic acid	C24H40O4	Terpenoids	11.81
Oroxylin A-7-O-beta-D-glucuronide	C22H20O11	Flavonoids	10.98
8-O-Acetylharpagide	C17H26O11	Iridoids	2.48
Chrysin	C15H10O4	Flavonoids	26.84
Baicalin	C21H18O11	Flavonoids	13.74
Galangin	C15H10O5	Flavonoids	8.29
Protocatechualdehyde	C7H6O3	Phenols	2.66
Quinic acid	C7H12O6	Organooxygen compounds	0.65
Jaceosidin	C17H14O7	Flavonoids	17.11
Caffeic Acid	C9H8O4	Phenylpropanoid	3.51
Fuziline	C24H39NO7	Terpenoids	11.5
Methyl hexadecanoate	C17H34O2	Fatty Acyls	12.23
7-Hydroxycoumarin	C9H6O3	Coumarins and derivatives	5.45
Tauroursodeoxycholic acid	C26H45NO6S	Terpenoids	8.75

Esculetin	C9H6O4	Phenylpropanoid	3.39
Genkwanin	C16H12O5	Flavonoids	11.41
Oroxylin A	C16H12O5	Flavonoids	7.71
Pristimerin	C30H40O4	Terpenoids	8.88
Biochanin A	C16H12O5	Flavonoids	10.4
Enoxolone	C30H46O4	Terpenoids	14.39
Glycocholic acid	C26H43NO6	Terpenoids	7.35
Oleanoic Acid	C30H48O3	Terpenoids	17.43
Alisol A 24-acetate	C32H52O6	Terpenoids	14.73
Emodin 8-O-(beta)-D-glucoside	C21H20O10	Quinones	7.22
Genistin	C21H20O10	Flavonoids	7.21
Kaempferol	C15H10O6	Flavonoids	6.91
HIPPURATE	C9H9NO3	Organic acids and derivatives	3.4
Shanzhiside	C16H24O11	Iridoids	1.46
Citric acid	C6H8O7	Carboxylic acids	0.82
D-Gluconic acid	C6H12O7	Organic acids and derivatives	0.66
7,8-Dihydroxycoumarin	C9H6O4	Phenylpropanoid	5.77
Kaempferol-3-O-glucoside	C21H20O11	Flavonoids	6.25
Secoisolariciresinol	C20H26O6	Phenylpropanoid	11.73
Maleic acid	C4H4O4	Organic acids and derivatives	0.77
Eupafolin	C16H12O7	Flavonoids	6.9
3-[(Carboxycarbonyl)amino]-L-alanine	C5H8N2O5	Alkaloids	28.51
Isopropyl 4-Hydroxybenzoate	C10H12O3	Phenolic acids	4.22
Linoleic acid	C18H32O2	Fatty Acyls	16.07
Gallic acid	C7H6O5	Phenols	0.96
Docosahexanoic acid	C22H32O2	Fatty acids	15.62
Gentisic acid	C7H6O4	Xanthones	2.5
Crocin	C44H64O24	Diterpenoids	8.87
Glutamylphenylalanine	C14H18N2O5	Carboxylic acids	3.27
CATECHOL	C6H6O2	Phenols	1.81
Ferulic acid	C10H10O4	Phenylpropanoid	5.44
arctiin	C27H34O11	Phenylpropanoid	7.01
Coumaroyl quinic acid	C16H18O8	Phenylpropanoid	4.22
geniposide	C17H24O10	Iridoids	4.22
Chlorogenic Acid	C16H18O9	Phenylpropanoid	3.14
Shanzhiside methyl ester	C17H26O11	Terpenoids	1.17
Deoxycholic acid	C24H40O4	Terpenoids	14.66
Medicagenic acid	C30H46O6	Terpenoids	12.04

Fusidine	C31H48O6	Terpenoids	12.35
Chenodeoxycholic acid	C24H40O4	Terpenoids	11.73
6,8-dihydroxy-2,2,4,4-tetramethyl-7-(3-methylbutanoyl)-9-(2-methylpropyl)-9H-xanthene-1,3-dione	C26H34O6	Terpenoids	10.53
Calcium pantothenate	C9H17NO5.1/2	Alkaloids	1.58
loganic acid	C16H24O10	Organic acids and derivatives	2.85
SUCCINATE	C4H6O4	Organic acids and derivatives	0.87
Ellagic acid	C14H6O8	Phenols	5.5
Spiculisporic acid	C17H28O6	Miscellaneous	11.68
4-Hydroxybenzoic acid	C7H6O3	Phenols	6.44
Quercetin	C15H10O7	Flavonoids	5.58
Abietic acid	C20H30O2	Terpenoids	13.23
kojic acid	C6H6O4	Organoheterocyclic	27.28
Quercetin-3-O-galactoside	C21H20O12	Flavonoids	3.55
Daidzein-8-C-glucoside	C21H20O9	Flavonoids	4.05
(2S,3S)-2-(3,4-dihydroxyphenyl)-3,7-dihydroxy-2,3-dihydrochromen-4-one	C15H12O6	Flavonoids	5.68
Genipin	C11H14O5	Iridoids	4.63
Quillaic acid	C30H46O5	Terpenoids	11.62
Glabrolide	C30H44O4	Terpenoids	9.13
Hastatoside	C17H24O11	Iridoids	2.55
Inermin	C16H12O5	Flavonoids	17
(EZ)-sinapic acid	C11H12O5	Phenylpropanoid	4.01
Inositol	C6H12O6	Organic oxygen compounds	0.6
Leucoside	C26H28O15	Flavonoids	5.8
13-HOTrE	C18H30O3	Organic acids and derivatives	16.06
Eleutheroside E	C34H46O18	Phenylpropanoid	5.47
Lamiide	C17H26O12	Iridoids	1.32
Protocatechuic acid	C7H6O4	Phenolic acids	1.8
cirsimarin	C23H24O11	Flavonoids	0.94
D-(+)-Malic acid	C4H6O5	Hydroxy acids and derivatives	1.65
Roburic acid	C30H48O2	Triterpenoids	16.16
Sorbose	C6H12O6	Benzene and substituted	3.06

Sinapic acid	C11H12O5	Phenylpropanoid	5.54
Azelaic acid	C9H16O4	Fatty Acyls	6.6
rhodioloside	C14H20O7	Phenols	2.58
Noreugenin	C10H8O4	Flavonoids	3.36
Glutaric acid	C5H8O4	Organic acids and derivatives	26.94
Genipin 1-O-beta-D-gentiobioside	C23H34O15	Iridoid glucoside	3.62
4-Methylcatechol	C7H8O2	Phenols	4.22
Ethyl myristate	C16H32O2	Aliphatic acyl	17.4
Corosolic acid	C30H48O4	Terpenoids	14.1
Deoxyloganic acid	C16H24O9	Prenol lipids	1.19
3-Phenyllactic acid	C9H10O3	Carbonyl	5.09
gamma-Linolenic acid	C18H30O2	Organic acids and derivatives	12.81
Schaftoside	C26H28O14	Flavonoids	5.8
isosakuranetin-7-O-neohesperidoside	C28H34O14	Flavonoids	7.21
Delphinidin	C15H11O7	Tetraterpenes	6.22
D(-)-Gulono-gamma-lactone	C6H10O6	Organic oxygen compounds	3.86
Sebacic acid	C10H18O4	Fatty Acyls	5.5
DL-beta-Hydroxybutyric acid	C4H8O3	Fatty Acyls	1.07
Kaempferol-7-O-rhamnoside	C21H20O10	Flavonoids	3.28
Propyl gallate	C10H12O5	Phenols	4.26
geniposidic acid	C16H22O10	Iridoid glucoside	2.65
sweroside	C16H22O9	Terpenoids	2.05
Eupatilin	C18H16O7	Flavonoids	10.86
Flavone base + 3O, C-Hex-dHex	C27H30O14	Flavonoids	5.46
4-Methoxysalicylic acid	C8H8O4	Phenols	1.46
13-HODE	C18H32O3	Lipids	16.74
Irigenin	C18H16O8	Flavonoids	9.86
Pectolinarigenin	C17H14O6	Flavonoids	10.28
Scutellarin	C21H18O12	Flavonoids	5.79
Sinapoylhexoside	C17H22O10	Phenylpropanoid	3.3
Methylsuccinic acid	C5H8O4	Fatty acids	22.71
3,4-Dihydroxyphenylethanol	C8H10O3	Phenols	4.88
Curcumin	C21H20O6	Phenols	2.45
FA 18:2+1O	C18H32O3	Miscellaneous	14.01
Herbacetin	C15H10O7	Flavonoids	5.36
Morin	C15H10O7	Flavonoids	6.5
osmanthuside H	C19H28O11	Flavonoids	2.99
Rhaponticin	C21H24O9	Phenols	1.17
Isobutyl 4-hydroxybenzoate	C11H14O3	Phenolic acids	7.3
Daidzin	C21H20O9	Flavonoids	4.88
santin	C18H16O7	Flavonoids	10.61

Tetrasaccharides	C24H42O21	Miscellaneous	6.24
Ascorbic acid	C6H8O6	Dihydrofurans	7.41
Feretoside	C17H24O11	Iridoids	2.55
Robinin	C33H40O19	Flavonoids	5.41
9-hydroxy-10,12-octadecadienoic acid	C18H32O3	Aliphatic acyl	13.02
12,13-EODE	C18H32O3	Fatty acids	12.76
Fraxetin	C10H8O5	Coumarins and derivatives	4.27
P-Anisic acid	C8H8O3	Phenols	3.41
Dehydrotumulosic acid	C31H48O4	Terpenoids	17.02
Dihydromyricetin	C15H12O8	Flavonoids	5.93
Mannitol	C6H14O6	Organooxygen compounds	1.3
4-[5-(4-hydroxy-3-methoxyphenyl)-3,	C20H24O5	Phenylpropanoid	12.02
Sucrose	C12H22O11	Carbohydrates and derivatives	0.59
Gentiopicroside	C16H20O9	Iridoids	3.06
Lipoic acid	C8H14O2S2	Organic heterocyclic	1.11
dihydroferulic acid	C10H12O4	Phenylpropanoids	2.07
Ecliptasaponin A	C36H58O9	Terpenoids	11.53
9-HODE	C18H32O3	Lipids	16.93
Arachidonic acid	C20H32O2	Fatty acids	17.03
Isochlorogenic acid B	C25H24O12	Phenylpropanoid	6.23
Bruceine A	C26H34O11	Terpenoids	7.05
Rosmarinic acid	C18H16O8	Phenylpropanoid	6.62
Flavone base + 3O, O-HexA-HexA	C27H26O17	Flavonoids	7.05
18alpha-glycyrrhetinic acid	C30H46O4	Terpenoids	16.43
PHENYLACETIC ACID	C8H8O2	Aromaticity	3.5
Isochlorogenic acid C	C25H24O12	Phenylpropanoid	6.54
Asperulosidic acid	C18H24O12	Iridoids	4.24
Coumarin + 1O + 1MeO, O-Hex-Hex	C22H28O14	Phenylpropanoid	4.97
Citraconic acid	C5H6O4	Fatty Acyls	3.87
Naringenin chalcone	C15H12O5	Flavonoids	7.4
